# Differentiation of two types of mobilized peripheral blood stem cells by microRNA and cDNA expression analysis

**DOI:** 10.1186/1479-5876-6-39

**Published:** 2008-07-22

**Authors:** Ping Jin, Ena Wang, Jiaqiang Ren, Richard Childs, Jeong Won Shin, Hanh Khuu, Francesco M Marincola, David F Stroncek

**Affiliations:** 1 Department of Transfusion Medicine, Clinical Center, National Institutes of Health, Bethesda, Maryland, USA; 2 Hematology Branch, National Heart, Lung, and Blood Institute, Bethesda, Maryland, USA; 3 Department of Laboratory Medicine, Soonchunhyang University Hospital, Seoul, Korea

## Abstract

**Background:**

Mobilized-peripheral blood hematopoietic stem cells (HSCs) have been used for transplantation, immunotherapy, and cardiovascular regenerative medicine. Agents used for HSC mobilization include G-CSF and the CXCR4 inhibitor AMD3100 (plerixafor). The HSCs cells mobilized by each agent may contain different subtypes and have different functions. To characterize mobilized HSCs used for clinical applications, microRNA (miRNA) profiling and gene expression profiling were used to compare AMD3100-mobilized CD133+ cells from 4 subjects, AMD3100 plus G-CSF-mobilized CD133+ cells from 4 subjects and G-CSF-mobilized CD34+ cells from 5 subjects. The HSCs were compared to peripheral blood leukocytes (PBLs) from 7 subjects.

**Results:**

Hierarchical clustering of miRNAs separated HSCs from PBLs. miRNAs up-regulated in all HSCs included hematopoiesis-associated miRNA; miR-126, miR-10a, miR-221 and miR-17-92 cluster. miRNAs up-regulated in PBLs included miR-142-3p, -218, -21, and -379. Hierarchical clustering analysis of miRNA expression separated the AMD3100-mobilized CD133+ cells from G-CSF-mobilized CD34+ cells. Gene expression analysis of the HSCs naturally segregated samples according to mobilization and isolation protocol and cell differentiation status.

**Conclusion:**

HSCs and PBLs have unique miRNA and gene expression profiles. miRNA and gene expression microarrays maybe useful for assessing differences in HSCs.

## Background

Hematopoietic stem cells (HSCs) have been used for more than 35 years for transplantation therapy to treat acute and chronic leukemia, lymphoma, marrow failure and congenital immune deficiency. Advances in immunotherapy have lead to the use of HSCs to produce dendritic cells (DCs) to enhance antigen presentation [1], to enhance leukocyte recovery after immunosuppresive therapy, and to mount cancer rejection by adoptive transfer of tumor infiltrating lymphocytes (TIL) [2]. HSCs have also been used to treat patients with ischemic cardiac disease to improve revascularization and cardiac function following acute myocardial ischemia [3,4]. However, due to the diversity of stem cell sources, mobilization methods employed, purity of cells, and the content of cell subsets, there are many different types of HSCs and those that are most beneficial for one application may not be best for another.

HSCs can be obtained from several different sources including bone marrow, mobilized peripheral blood, and umbilical cord blood. For transplantation, traditionally, HSCs were obtained from the bone marrow. However, umbilical cord blood has been found to be especially rich in HSCs [5] and HSCs have been found in the peripheral blood and their level in the circulation increases several-fold after G-CSF administion [6,7]. For HSC transplantation all three types of HSCs are used, but for most other applications mobilized peripheral blood HSCs are most commonly used.

The diversity of HSCs used for clinical therapies has also increased due to the development of new HSC mobilizing agents. For many years granulocyte colony-stimulating factor (G-CSF) has been the standard agent to increase the level of circulating HSCs. The administration of G-CSF daily for 4 to 6 days results in a 10- to 30-fold increase in the number of circulating HSCs [8,9] and G-CSF-mobilized HSCs collected by apheresis have been used for transplantation, immune therapy and the treatment of cardiac ischemia. Another HSC mobilizing agent, AMD3100, has been used with G-CSF to mobilize stem cells for autologous transplants [10] and is currently being evaluated as a single agent to mobilize HSCs for allogeneic donor transplants [11,12].

The mechanisms by which AMD3100 and G-CSF alter HSC trafficking and mobilization are different suggesting that HSCs with different intrinsic properties maybe be mobilized by these agents. AMD3100, as a CXCR4 antagonist, mobilizes HSCs within 6 hours by disrupting the engagement of stem cell surface CXCR4 with its ligand SDF-1 (CXCL12) which is expressed on marrow osteoblasts [10,13-20]. In contrast G-CSF mobilizes stem cells indirectly by down regulating the expression of SDF-1 on marrow osteoblasts and by releasing neutrophil and monocyte proteolytic enzymes including neutrophil elastase, cathepsin G, and maxtrix metalloproteinase-9 which in turn degrade important HSC trafficking and adhesion molecules c-kit, VCAM-1, CXCR4, and SDF-1 [21]. In animal studies AMD3100 mobilizes a CD34+ cell population with a greater long-term marrow repopulating capacity than G-CSF [12,22,23], possibly due to differences in mechanisms of mobilization.

Although commonly accepted HSC specific surface markers have been used for HSC characterization and purification, differences in the specificities of monoclonal antibodies used to isolate HSCs have contributed to diversity in HSC clinical products. Antibodies specific for CD34 have been the standard agent for the isolation of HSCs. In addition, anti-CD133 has also been used [24-27]. Approximately 75% of G-CSF mobilized peripheral blood stem cells (PBSCs) express CD34 as well as CD133, but small populations express one or the other [28].

MicroRNAs (miRNA) are short, 20–22 nucleotide long, RNA molecules which negatively regulate protein translation in a variety of biological processes, including developmental timing, signal transduction, tissue differentiation and stem cell renewal and differentiation. Some miRNAs are specifically expressed in stem cells and control stem cell self-renewal and differentiation by negatively regulating the expression of certain key genes in stem cells.

To determine if miRNA and gene expression profiling would be beneficial in distinguishing different types of HSCs, we compared CD133+ cells isolated from AMD3100- and AMD3100 plus G-CSF-mobilized PBSC concentrates with CD34+ cells isolated from G-CSF-mobilized PBSC concentrates. We applied miRNA profiling and gene expression profiling analysis to assess these three different types of progenitor cell populations using peripheral blood T cells, B cells, monocytes and NK cells as a reference. We hypothesized that miRNA and gene expression analysis would be useful for characterizing HSCs. Global gene and miRNA expression profiling was used to compare HSCs and peripheral blood leukocytes. T cells, B cells, monocytes and NK cells isolated from peripheral blood mononuclear cells (PBMCs) were compared to the HSCs.

## Methods

### Hematopoietic progenitor cell isolation

#### AMD3100 and AMD3100 plus G-CSF stem cell mobilization, collections and isolation

For the mobilization of cells with AMD3100 alone, one dose of AMD3100 (Plerixafor, Genzyme Corporation, Cambridge, MA) (240 μg/kg) was given subcutaneously and PBSC concentrate was collected by leukapheresis (CS3000, Baxter Healthcare Corp., Fenwal Division, Deerfield, IL) six hours later. For AMD3100 plus G-CSF-mobilization G-CSF (10 μg/kg) (Filgrastim, Amgen, Thousand Oaks, CA) was given subcutaneously to healthy subjects daily for four days and on the fifth day in addition to G-CSF, AMD3100 (240 μg/kg) was given subcutaneously. A mobilized PBSC concentrate was collected by leukapheresis (CS3000, Baxter Healthcare Corp.) on day 5 twelve hours after the administration of AMD3100 and 2 hours after the last dose of G-CSF. CD133+ cells were isolated from the PBSC concentrates by positive selection with anti-CD133 and magnetic microparticles (CliniMacs, Miltenyi Biotec, Bergisch Gladbach, Germany) and the isolated cells were cryopreserved. The proportion of isolated cells that expressed CD133 ranged from 88% to 98%.

#### G-CSF stem cell mobilization, collection, and isolation

Healthy subjects were given G-CSF (10 μg/kg) (Filgrastim, Amgen Thousand Oaks, CA) subcutaneously daily for 5 days. A mobilized PBSC concentrate was collected by leukapheresis (CS3000, Baxter Healthcare Corp) on the fifth day and cryopreserved in 5% DMSO and 6% pentastarch using a controlled rate freezer and stored in liquid nitrogen. The PBSC concentrate was thawed and CD34+ cells were isolated by immunoaffinity chromatography with CD34 monoclonal antibody and magnetic beads (Isolex, Baxter Healthcare). The proportion of isolated cells that expressed CD34 ranged from 87% to 98%.

### Non-mobilized PBMC collection and sample preparation

A non-mobilized PBMC concentrate was collected from 7 healthy subjects by apheresis (CS3000) at the Department of Transfusion Medicine (DTM), Clinical Center (CC), National Institutes of Health (NIH). All subjects signed an informed consent approved by the NIH. Mononuclear leukocytes were separated from contaminating granulocytes and red blood cells by Ficoll gradient separation, cryopreserved and stored in liquid nitrogen. T cells, B cells, NK cells and monocytes were isolated by positive selection by using anti-CD3, anti-CD19, anti-CD56 and anti-CD14 magnetic beads, respectively (AutoMACS, Miltenyi Biotec) (purity ≥ 90%).

### RNA preparation, amplification and labeling

Total RNA was isolated from each sample by using TRIZOL reagent (Invitrogen, Carlsbad, CA).

#### cDNA expression array

Total RNA (3 μg) was amplified from 0.5 × 10^6 ^to 10^7 ^cells into anti-sense RNA (aRNA), also, total RNA from PBMCs pooled from six normal donors was extracted and amplified into aRNA to serve as the constant reference [29] Test and reference RNAs were labeled with Cy5 (red) and Cy3 (green) dyes, respectively, and co-hybridized to the custom-made 17.5 K cDNA (UniGene cluster) microarrays which were printed in the Immunogenetics Section of DTM with a configuration of 32 × 24 × 23 [30]. Clones used for printing included a combination of the Research Genetics RG_HsKG_031901 8 k clone set and 9,000 clones selected from the RG_Hs_seq_ver_070700 40 k clone set. The 17,500 spots included 12,072 uniquely-named genes, 875 duplicated genes and about 4,000 expression sequence tags. The complete list of genes included in the Hs-CCDTV-17.5k-1px printing is available at the web site .

#### MicroRNA array

A miRNA probe set was designed using mature antisense miRNA sequences (Sanger data base, version 8.1) consisting of 736 human, mouse, rat and virus plus two control probes. The probes were 5' amine modified and printed in duplicate in the Immunogenetics Section of the DTM on CodeLink activated slides (General Electric, GE Health, NJ, USA) via covalent bonding.Small RNA was enriched from 10 ug total RNA by flashPAGE (Pre-cast Gel, Type A) (Ambion, Austin, TX, USA) and purified using flashPAGE reaction clean-up kit (Ambion, Austin, TX, USA) according to manufacture's instruction. The same procedures were applied to obtain small RNA from the Epstein-Barr virus (EBV)-transformed lymphoblastoid cell lines that was used as the reference for the miRNA expression array assay. Fragmented small RNA were 3'-end tailed with amine-modified nucleotides and chemically coupled to CyDye fluors (Amersham Biosciences, piscatway, NJ, USA), the test sample with Cy5 and the reference with Cy3, using the mirVana miRNA Labeling Kit (Ambion) following the manufacturer recommended protocol. After labeling, the samples and the reference were co-hybridized to the miRNA array at room temperature over night. Both the processed cDNA and the miRNA array slides were scanned by GenePix scanner Pro 4.0 (Axon, Sunnyvale, CA, USA).

### Data and statistical analyses

The raw microarray data set was filtered according to a standard procedure to exclude spots with minimum intensity that was arbitrarily set to an intensity parameter of ≥ 300 for cDNA expression data and ≥ 100 for the miRNA microarray data in both fluorescence channels. If the fluorescence intensity of one channel was below the cut-off while the other was above, the lower channel intensity was overridden. Spots with diameters < 25 μm for cDNA expression array and < 10 μm for miRNA microarry and flagged spots were also excluded from the analyses. Then, the filtered data were normalized using Lowess Smoother and retrieved by the BRB ArrayTool  developed at the National Cancer Institute (NCI), Biometric Research Branch, Division of Cancer Treatment and Diagnosis. Hierarchical cluster analysis and TreeView software [31] were used for visualization [32]. All of the predictions of miRNA gene targets were made using BRB ArrayTool microRNA targets  developed at the NCI, Biometric Research Branch, Division of Cancer Treatment and Diagnosis.

## Results

### Peripheral blood leukocytes and hematopoietic progenitor cells

PBMC concentrates were collected by apheresis from 7 healthy subjects. B cells, T cells, NK cells, and monocytes were isolated from each PBMC concentrate. 92% to 98% of the isolated B cells expressed CD21, 95% to 98% of T cells expressed CD3, 90% to 97% of NK cells expressed CD56 and 92% to 98% of monocytes expressed CD14. Mobilized peripheral blood HSCs were collected from 13 healthy subjects: 5 were given G-CSF and their HSCs isolated with anti-CD34 while 4 were given AMD3100 alone and 4 were given AMD3100 plus G-CSF and their HSCs isolated with anti-CD133. In the four donors given AMD3100 plus G-CSF the concentration of circulating CD34+ cells increased from 62 ± 45 × 10^6^/L prior to the administration of AMD3100 to 215 ± 117 × 10^6^/L twelve hours after AMD3100 was given, suggesting that AMD3100 was responsible for mobilizing a substantial portion of the circulating CD34+ cells. In the four donors given AMD3100 the CD34+ cell counts increased from a baseline level of 3 ± 4 × 10^6^/L to 23 ± 4 × 10^6^/L 6 hours after the administration of AMD3100.

### Comparison of miRNA expression among hematopoietic progenitor cells and peripheral blood leukocytes

**Figure 1:**
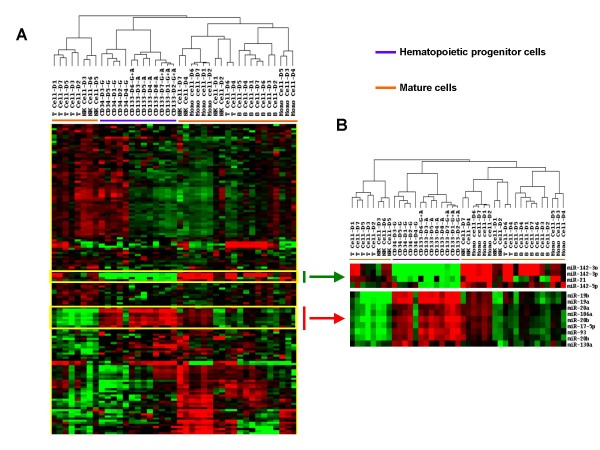
**MicroRNA (miRNA) expression profiles of hematopoietic stem cells (HSCs) and peripheral blood leukocytes (PBLs)**. RNA was isolated from T cells, B cells, monocytes and NK cells from 7 subjects, G-CSF-mobilized CD34+ cells from 5 subjects, AMD3100 (A)-mobilized CD133+ cells from 4 subjects, and AMD3100 plus G-CSF (A+G)-mobilized CD133+ cells from 4 subjects and miRNA expression was analyzed using an expression array with 457 human miRNAs. A) Unsupervised hierarchical clustering of Eisen was used to analyze the 148 miRNAs that remained after filtering (miRNA expressed in ≥ 80% of samples). B) Signature miRNAs whose expression was markedly up-regulated in HSCs or PBLs.

Among the 13 HSC samples and the 28 peripheral blood leukocyte (PBL) samples analyzed ≥ 80% of the samples expressed 148 miRNAs of the 457 human miRNA in our chip. Unsupervised hierarchical clustering analysis based on the 148 miRNAs revealed 3 distinct groups: the exclusively HSC cluster; the T (n = 5) and NK cell (n = 3) cluster; and the B cell and monocyte dominant cluster which contained a mixture of B cells (n = 7), monocytes (n = 7), T cells (n = 2) and NK cells (n = 4) (Figure 1A). Within the HSC group the 4 AMD3100-mobilized and the 4 AMD3100 plus G-CSF-mobilized CD133+ cells clustered together, but separate from the G-CSF-mobilized CD34+ cells.

The make-up of the miRNAs that separated HSCs and PBLs differed markedly. Eight signature miRNAs demonstrated increased expression and 3 miRNAs decreased expression in HSCs compared to PBLs (Figure 1B). The 8 signature HSC miRNAs whose expression was increased included miR-19a, -19b, -20a, and 20b, which are part of the polycistronic cancer-associated miR-17-92 cluster. The 3 signature miRNAs whose expression was greatest in the PBL samples were miR-142-3p, miR-21, and miR-142-5p. Both probes for miR-20b were up-regulated in HSCs and both for miR-142-3p were up-regulated in PBLs.

To identify the entire set of miRNAs whose expression differed between HSCs and PBLs, the expression of miRNAs by all 13 HSC samples was compared with those by all 28 PBL samples (t-test, p < 0.005). The expression of 35 miRNAs differed between the two types of cells: the expression of 13 miRNAs were increased in HSCs and 22 miRNAs were increased in PBLs (Table 1). The expression of two miRNAs, miR-126 and miR-10a, were markedly increased in HSCs. Both miR-126 and miR-10a and 7 others, miR-19a, -19b, -17-5p, 20b, -93, -130a and -221, have been previously reported to be expressed by HSCs [33-35]. Among the 13 miRNAs whose expression was increased in HSCs were 3 miRNAs belonging to the cancer-associated miR-17-92 cluster; miR-19a, 19b, and -17-5p. When the expression of all the miRNAs in the miR-17-92 cluster (n = 7) were compared between HSCs and PBLs, 5 of the 7 were increased in HSCs (Table 2). Regulated by the oncogene cMyc, the miR-17-92 cluster targets large numbers of genes. Consistent with this notion, the expression of cMyc gene was increased 3.3-fold in HSCs based on our gene expression analysis. The expression of 3 miRNAs in a second cancer-associated miR-106-303 cluster were also increased in HSCs (Table 2). In contrast, expression of two cancer suppressing miRNAs, miR-15a and miR-16, were decreased in HSCs.

**Table 1:** MicroRNAs (miRs) whose expression differed among 13 hematopoietic stem cell

**miRs whose expression was** ** increased in HSCs**	**Fold-increase**	**miRs whose expression** ** was increased in PBLs**	**Fold-increase**
hsa-miR-126	14.43	hsa-miR-142-3p	15.66
hsa-miR-10a	13.46	hsa-miR-218	11.07
hsa-miR-19a	3.89	hsa-miR-21	8.46
hsa-miR-19b	3.11	hsa-miR-379	7.63
hsa-mir-595	2.98	hsa-miR-381	4.61
hsa-miR-146a	2.72	hsa-miR-29b	3.83
hsa-miR-93	2.56	hsa-miR-26b	3.54
hsa-miR-221	2.38	hsa-miR-30c	3.12
hsa-miR-20b	2.35	hsa-miR-142-5p	2.51
hsa-miR-130a	2.25	hsa-miR-29a	2.49
hsa-miR-34a	2.12	hsa-let-7g	2.49
hsa-miR-363	1.92	hsa-let-7i	2.42
hsa-miR-17-5p	1.89	hsa-miR-191	2.3
		hsa-miR-30b	2.03
		hsa-let-7b	1.88
		hsa-miR-26a	1.85
		hsa-miR-16	1.78
		hsa-let-7c	1.75
		hsa-miR-30a-5p	1.73
		hsa-miR-373	1.62
		hsa-mir-594	1.6
		hsa-mir-610	1.34

(HSC) and 28 peripheral blood leukocyte (PBL) samples*

* The expression of miRs between the two groups were compared with t-tests (p < 0.005)

**Table 2:** Cancer-associated microRNAs (miRNAs) whose expression was up- and down-regulated in hematopoietic stem cells (HSCs)

**Cluster miR-17-92**		
**miRNA**	**Fold-increase in HSCs**	**P**

17-p5	1.89	0.004
17-p3	NC	NS
18a	1.31	0.018
19a	3.89	4 × 10^-7^
20a	1.55	0.015
19b-1	3.1	1.3 × 10^-6^
92-1	NC	NS

**Cluster miR-106-363**		

**miRNA**	**Fold-increase in HSCs**	**P**

106a	1.55	0.016
18b	NC	NS
20b	2.34	2.9 × 10^-05^
19b-2	3.1	1.3 × 10^-06^
92-2	NC	NS
363	NC	NS

**Cluster miR-15-16**		

**miRNA**	**Fold-decrease in HSCs**	**P**

15a	3.33	0.007466
16	1.78	9.93 × 10^-4^

NC = no change

NS = not significant

Among the 148 miRNAs 47 were differentially expressed between AMD3100-mobilized CD133+ cells and the G-CSF-mobilized CD34+ cells (t-tests, p < 0.005). The expression of 17 was increased in the AMD3100 group and 30 were increased in the G-CSF group (data not shown).

The expression of 4 miRNAs were markedly increased in PBL: miR-142-3p, miR-218, miR-21, and miR-379. In addition to these 4 miRNAs, expression of miR-16 which belongs to the leukemia associated cluster miR-15a-16 was also increased.

To identify miRNAs uniquely expressed among PBLs, 5-way ANOVA was performed to compare the expression of the 148 miRNAs among T cell, B cell, monocyte, NK cell and the HSC samples. T cells were characterized by increased expression of miR-146a, -146b, -29a, and -29b and B cells were characterized by the increased expression of miR-29a -29b, and -29c (Table 3). The expression of several miRNAs including miR-223 and miR-21 were increased in monocytes, but none were increased NK cells (Table 3).

**Table 3:** MicoRNAs (miRNAs) characteristic of each type of peripheral blood leukocyte*

**T cells**				**NK cells**			
**Increased**	**FC**	**Decreased**	**FC**	**Increased**	**FC**	**Decreased**	**FC**

miR-146b	4.05	miR-223	16.8	None	NA	miR-19a	3.54
miR-29b	3.86	miR-181b	2.41			miR-19b	3.06
miR-146a	3.45	miR-93	2.32			miR-92	1.85
miR-29a	3.38	miR-20b	2.19				
		miR-17-5p	2.01				
		miR-20a	1.88				
		miR-106a	1.86				

**Monocytes**				**B cells**			

**Increased**	**FC**	**Decreased**	**FC**	**Increased**	**FC**	**Decreased**	**FC**

miR-223	21.0	miR-146b	5.85	miR-29b	4.53	miR-22	7.62
miR-21	10.1	miR-146a	5.71	miR-29a	3.66	miR-23a	6.15
miR-424	5.74	miR-29c	2.92	miR-29c	3.05	miR-24	5.87
miR-365	3.28					miR-146b	3.33
miR-191	3.28					miR-27a	3.29
miR-103	3.19					miR-23b	2.42
miR-23a	3.1						
miR-27a	2.85						
miR-15a	2.77						
miR-374	2.74						
miR-107	2.58						
miR-106b	2.33						
miR-16	2.1						
miR-422b	2.01						
miR-23b	1.78						
miR-185	1.62						

*The groups of cells were compared using F-tests (p < 0.005)

FC = fold change

### cDNA expression profiling

**Figure 2:**
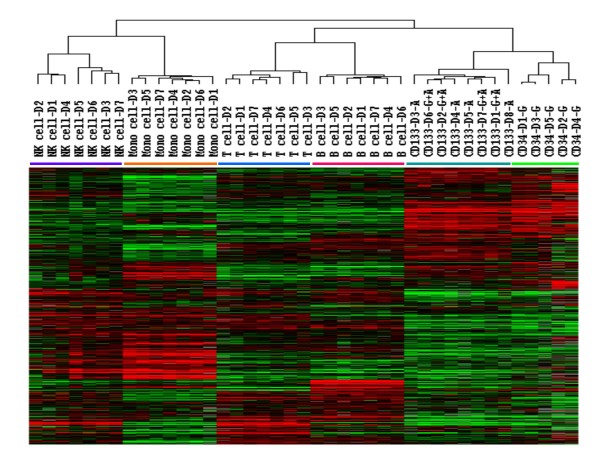
**Gene expression profiles of hematopoietic stem cells (HSCs) and peripheral blood leukocytes (PBLs)**. cDNA was isolated from T cells, B cells, monocytes and NK cells from 7 subjects, G-CSF (G)-mobilized CD34+ cells from 5 subjects, AMD3100 (A)-mobilized CD133+ cells from 4 subjects and AMD3100 plus G-CSF (A+G)-mobilized CD133+ cells from 4 subjects. cDNA expression was analyzed using an expression microarray with 17,500 cDNA. Unsupervised hierarchical clustering of Eisen was used to analyze the 11,023 genes that remained after filtering (cDNA expressed in ≥ 80% of samples).

Analysis of miRNA expression revealed that HSCs and PBLs had unique miRNA expression profiles. To investigate this further, gene expression profiles were also compared using cDNA expression microarrays which contained 17,088 genes. Unsupervised hierarchical clustering of the 11,023 genes that were expressed in ≥ 80% of the samples separated the HPC and PBL samples into two distinct groups (Figure 2). All of the HSC samples clustered separately from mature PBL subsets. The AMD3100-mobilized CD133+ cells and the AMD3100 plus G-CSF-mobilized CD133+ cells clustered together and again were considered as one group, the AMD3100 group. The two different types of HSCs, AMD3100-mobilized CD133+ cells and G-CSF-mobilized CD34+ cells clustered separately (Figure 2). In addition, T cell, B cell, monocyte, and NK cell samples clustered into separate groups (Figure 2).

A comparison of genes expressed by HSCs and PBLs revealed that 5,392 genes were differentially expressed among the two types of cells (t-tests, p < 0.001). The 30 genes whose expression increased the greatest in HSCs included several transcription factors and oncogenes such as GATA2 and N-myc, while the 30 genes whose expression was increased the most in PBLs were enriched for genes known to be expressed by circulating leukocytes such as Fc-γ receptor III (CD16), integrin α M subunit and IL-10 receptor α (Table 4).

**Table 4:** Genes differentially expressed between hematopoietic stem cells (HSCs) and peripheral blood leukocytes (PBLs)

**Expression Increased in HSCs**			**Expression Increased in PBLs**		
**Gene Symbol**	**Description**	**Fold Change**	**Gene Symbol**	**Description**	**Fold Change**

GATA2	GATA-binding protein 2	237.82	CARD14	Caspase recruitment domain protein 14	25.59
MYCN	N-myc	97.75	LTBR	Lymphotoxin-Beta receptor precursor = Tumor necrosis factor receptor 2 related protein = Tumor necrosis factor C receptor	18.83
CRHBP	CRF-BP = corticotropin-releasing factor binding protein	65.52	GPR65	TDAG8 = putative G protein-coupled receptor induced during activation-induced apoptosis of T cells = G protein-coupled receptor 65	18.33
FHL1	Four and a half LIM domains 1	58.2	FCGR3A	CD16 = Fcgamma receptor IIIa	18.15
ERG	V-ets erythroblastosis virus E26 oncogene homolog (avian)	43.57	ITGAM	Integrin, alpha M (complement component 3 receptor 3 subunit)	16.96
NPR3	Natriuretic peptide receptor C/guanylate cyclase C (atrionatriuretic peptide receptor C)	42.04	SGSH	N-sulfoglucosamine sulfohydrolase (sulfamidase)	16.64
SCHIP1	Schwannomin interacting protein 1	41.77	ITGB7	Integrin, beta 7	16.39
MSRB3	Methionine sulfoxide reductase B3	35.29	GNLY	Granulysin	15.21
MYB	V-myb myeloblastosis viral oncogene homolog (avian)	32.99	ALOX5AP	Arachidonate 5-lipoxygenase-activating protein	15.11
DEPDC6	DEP domain containing 6	28.85	CD48	CD48 = BLAST-1	14.81
EPDR1	Ependymin related protein 1 (zebrafish)	27.88	IL10RA	Interleukin 10 receptor, alpha	13.71
TRH	Thyrotropin-releasing hormone	26.63	CX3CR1	Chemokine (C-X3-C motif) receptor 1	13.64
NGFRAP1	Nerve growth factor receptor (TNFRSF16) associated protein 1	25.86	COTL1	Coactosin-like 1 (Dictyostelium)	13.4
SERPING1	Serpin peptidase inhibitor, clade G (C1 inhibitor), member 1, (angioedema, hereditary)	25.83	ADAM19	ADAM metallopeptidase domain 19 (meltrin beta)	13.24
MAP7	Microtubule-associated protein 7	25.11	IL2RB	IL-2 receptor beta chain	13
SOCS2	Suppressor of cytokine signaling 2	24.44	ITK	IL2-inducible T-cell kinase	11.91
TSC22D1	TSC22 domain family, member 1	24.12	KLRC4	Killer cell lectin-like receptor subfamily C, member 4	11.36
H1F0	H1 histone family, member 0	22.71	CCL4	MIP-1 beta	11.22
RBPMS	RNA binding protein with multiple splicing	22.67	CTSH	Cathepsin H	11.18
CTDSPL	CTD (carboxy-terminal domain, RNA polymerase II, polypeptide A) small phosphatase-like	22.64	EBI2	Epstein-Barr virus induced gene 2 (lymphocyte-specific G protein-coupled receptor)	10.69
CDCA7	Cell division cycle associated 7	22.49	GIMAP4	GTPase, IMAP family member 4	10.28
CYTL1	Cytokine-like 1	20.9	POU2F2	POU domain, class 2, transcription factor 2	10.22
FLT3	Fms-related tyrosine kinase 3	20.45	CXCR4	CXCR4 = CXC chemokine receptor 4	10.11
PRKAR2B	Protein kinase, cAMP-dependent, regulatory, type II, beta	20.09	CYP4A11	Cytochrome P450, family 4, subfamily A, polypeptide 11	10.1
TRIM58	Tripartite motif-containing 58	19.18	LGALS3	Lectin, galactoside-binding, soluble, 3 (galectin 3)	9.61
FSCN1	Fascin homolog 1, actin-bundling protein (Strongylocentrotus purpuratus)	18.91	SNX27	Sorting nexin family member 27	9.44
C1orf150	Chromosome 1 open reading frame 150	18.83	TRIM26	Tripartite motif-containing 26	9.23
TRH	Thyrotropin-releasing hormone	17.56	SNX27	Sorting nexin family member 27	9.05
KIT	V-kit Hardy-Zuckerman 4 feline sarcoma viral oncogene homolog	17.27	CSPG2	Chondroitin sulfate proteoglycan 2 (versican)	8.89
WASF1	WAS protein family, member 1	16.75	IL32	Interleukin 32	8.76

*The groups were compared using t-tests (p < 0.001).

## Discussion

In this study we discovered that miRNA expressed by HSC differed from those expressed by PBLs. We also found that miRNA profiling and cDNA expression analysis are potentially useful tools for the analysis of clinical HSCs derived by different mobilization and isolation methods which may yield functionally diverse products.

Among the 13 miRNAs increased in HSCs miR-10a, -17-5p, -19a, -19b, -20b, -93, -126, -130a, and -221; have previously been found to be expressed by HSCs [33-36]. miR-126 and miR-10a have been found to be down-regulated in erythrocyte and megakaryocyte precursors [33,36]. miR-221 has previously been shown to be important in erythropoiesis; the expression of miR-221 and -222 inhibit erythropoiesis [37]. The expression of miR-221 has been reported to be down regulated in CD34+ cells during erythropoesis [33,37]. The expression of miR-221 is also increased in papillary thyroid carincomas [38] and is involved with the pathogenesis of hepatocelluar carincoma [39] and prostate cancer [40].

Polycistronically transcribed miRNA clusters, so called onco-miR clusters, were also highly expressed by HSCs compared to PBLs. These findings are consistent with the theory that stem cells are important in cancer. The miR-17-92 cluster has been reported to be up-regulated in diffuse large B cell lymphoma (DLBCL), lung, breast, prostate, and colon cancer [41-43]. Venurini and colleagues found that the miR-17-92 cluster was up-regulated in CD34+ cells from healthy subjects and those with early-chronic phase chronic myelogenous leukemia, but was not up-regulated in CD34+ cells from subjects with CML blast crisis [44]. In addition, 3 of 6 members of the cancer-associated miR-106-363 cluster were also up-regulated in HSCs. Elevated expression of this cluster has been found in T cell leukemia [45] and T cell lymphoma [46].

miR-21 was increased in PBLs. miR-21 has also been found to be increased in chronic lymphocytic leukemia [47], B cell lymphomas [48], breast cancer [49], pancreatic cancer [50], head and neck cancer cell lines [51]. In addition, the expression of miR-16 was greater in PBLs, especially in monocytes, than HSCs. miR-15 and miR-16 are cancer-associated miRNA that are down-regulated in B-cell chronic lymphocyte leukemia [52,53] and pituitary adenomas [54] but upregulated in acute promyelocytic leukemia [55]. We also found that T cells were characterized by the upregulation of miR-29a and -29b and B cells by miR-29a, -29b, and -29c. These miRNA are down-regulated in aggressive B cell lymphoma [56].

At the transcription level we found that the expression of a number of oncogenes and genes related to transcription was greater in HSCs than in PBLs. The expression of GATA2, a transcription factor important in hematopoietic stem cell and endothelial cell differentiation [57,58] was 137-fold greater in HSCs and that of N-myc was 97-fold greater. Computational gene target prediction  indicates that GATA2 is targeted by miR-27a whose expression was 2-fold less in HSCs than PBLs (p = 0.0069).

An interesting finding of this study resides in the differences in both miRNA and gene expression found between G-CSF-mobilized CD34+ cells and AMD3100-mobilized CD133+ cells. While both miRNA expression profiling and DNA expression profiling differentiated the two different types of HSCs, these two populations were obtained using different HSC mobilization and isolation procedures. It is not certain if the differences in the two stem cell types were due to the different mobilizing agents or isolation antibodies. We suspect that both the mobilizing agents and antibodies contributed to the differences. In addition, the G-CSF-mobilized CD34+ cells underwent an additional freeze-thaw cycle. This was not expected to affect the HSCs, but we can not exclude this possibility. Further studies are needed which compare HSCs mobilized with AMD3100 alone and with G-CSF alone and with both types of stem cells isolated with the same monoclonal antibody. Since both CD34 and CD133 are being used to isolate stem cells for clinical applications, HSCs mobilized with one agent and isolated with each antibody should also be compared.

When considering new or different mobilization or isolation protocols, changes in both the quantity and quality of HSCs should be considered. The quantitation of CD34+ cells has been the gold standard for assessing the potency of HSCs for clinical therapies. When comparing HSCs mobilized with G-CSF from different subjects, different G-CSF mobilization protocols, or different HSC collection protocols, measuring CD34+ cells is a good indicator of the potency of the HSCs. However, if stem cells mobilized with G-CSF are to be compared with those mobilized with AMD3100 or AMD3100 plus G-CSF, measuring only CD34+ or CD133+ cells may not completely reflect the differences among these types of cells. Measures in addition of CD34 and CD133 are needed to more completely characterize HSCs.

Our results show that the global miRNA and cDNA expression assessment can distinguish different types of HSCs. Both types of global gene expression arrays were able to distinguish the two types of HSCs that we studied and both should be useful for potency testing of clinical HPC components. miRNA and cDNA microarray assays with several thousand probes usually require several days to complete, but it would be possible to more rapidly analyze the expression of a smaller selected group of miRNA or genes.

In conclusion, HSCs and PBLs have unique miRNA expression profiles and many cancer-associated miRNA are expressed by HSCs. miRNA and gene expression microarrays maybe useful for assessing differences in HSCs.

## Competing interests

The authors declare that they have no competing interests.

## Authors' contributions

All of the authors read and approved the final manuscript. The studies were designed by PJ and DS and were preformed by PJ, JR, and JWS. Stem cell mobilization and isolation was performed under the direction of RC and HK. The data was analyzed by PJ, EW, DS and FM. PJ and DS wrote the manuscript with help from RC and EW.
